# Task-Specific and Latent Relationships Between Motor Skills and Executive Functions in Preschool Children

**DOI:** 10.3389/fpsyg.2020.02208

**Published:** 2020-09-18

**Authors:** Gerda Van Der Veer, Erica Kamphorst, Marja Cantell, Alexander Minnaert, Suzanne Houwen

**Affiliations:** Department of Special Needs Education and Youth Care, University of Groningen, Groningen, Netherlands

**Keywords:** motor skills, executive functioning, inhibition, working memory, cognitive flexibility, early childhood, factor structure

## Abstract

There has been an increasing interest in the relationship between motor skills and executive functions (EFs) in young children over the years. However, no clear picture on the relationship between both domains has emerged from these studies. We have extended previous findings by conducting a comprehensive examination of task-specific and latent relationships between a range of motor skills and EFs in preschool children. The sample consisted of 198 3- to 5-year-old children (102 boys; 51.5%). Motor skills were assessed using the Movement Assessment Battery for Children Second Edition. EFs were assessed with the performance-based tasks ‘Day/Night,’ ‘Hand Tapping,’ ‘Forward Corsi Block,’ ‘Forward Digit Recall,’ and ‘Conflict Task,’ and a rating-based EF measure (i.e., the Behavior Rating Inventory of Executive Functioning - Preschool version). Task-specific relationships were examined using zero-order Pearson correlations. Latent factors of motor skills and EFs were examined using confirmatory factor analysis and exploratory structural equation modeling. Structural equation modeling (SEM) was used to examine latent relationships. The results of the Pearson correlation analyses showed statistically significant albeit weak correlations between specific motor and EF items (*r* = 0.15 to *r* = *0.23*). SEM showed non-significant weak relationships between a general motor factor (as a unitary latent construct) on the one hand, and performance-based EFs and rating-based EFs (as latent EF components) on the other hand. In conclusion, this study suggested only weak relationships between motor skills and EFs in preschool children with no clear differences between their task-specific and latent relationships.

## Introduction

Motor skills undisputedly play an important role in peoples’ overall functioning. This is especially true for young children as the attainment of motor skills provides children with new opportunities for learning about their physical and social environment, both regarding objects and other individuals (Wilson, 2002; Adolph and Joh, 2007; von Hofsten, 2009). This theoretical claim about a relationship between the motor and cognitive domains, more specifically the relationship between motor skills and executive functions (EFs) has attracted the attention of many early childhood researchers over the years. Thus far, research has not been able to elucidate a clear picture on the relationship between these two domains (Livesey et al., 2006; Michel et al., 2011; Lehmann et al., 2014; Houwen et al., 2017; Oberer et al., 2017; Alesi et al., 2019; Maurer and Roebers, 2019), as studies have used different and sometimes limited sets of only one or two motor skills and/or EFs. In addition, previous studies have examined the relationship between motor skills and EFs on exclusively one level (i.e., on an item-level or a construct-level), making it difficult to gain insight into the multi-level nature of the relationship. It is important to know whether and to what extent a relationship between motor skills and EFs exists on an item-level and/or on a construct-level as this information may have implications for the design of early interventions. More specifically, it provides information whether interventions should be focused on the domains in general or on specific motor skills and/or EFs. For example, if a relationship exists between a specific motor task (e.g., threading beads) and a specific inhibition task (e.g., a Stroop task) without relationships between other specific fine motor and inhibition tasks, then interventions should focus on specific tasks without the expectation of a generic effect on the performance of other motor and EF tasks. On the other hand, if a relationship is found between fine motor skills and inhibition on a more general level (i.e., on a construct-level), then interventions can focus on several fine motor and/or inhibition tasks with the expectation of more generic effects on the performance of other fine motor and inhibition tasks as well. Therefore, this study extends previous research by examining both task-specific and latent relationships in a range of motor skills and EFs in order to provide more insight into the multi-level nature of the relationship between motor skills and EFs in preschool children.

From a theoretical point of view, there are several explanations for a relationship between motor skills and EFs. The embodied cognition theory suggests that cognition, including EFs, is grounded in motor development (Foglia and Wilson, 2013). Motor development provides children new opportunities for actively exploring their physical and social environment through ongoing perception-action cycles, which supports cognitive development (von Hofsten, 2007). The acquisition of new cognitive capacities, in turn, allows for the acquisition of more varied and complex motor skills (Adolph and Hoch, 2019). In a similar vein, the concept of reciprocity and the theory of automaticity have been put forward as being useful in understanding the active and ongoing interaction of motor and EF development (Kim et al., 2018; McClelland and Cameron, 2019). Reciprocity occurs when skills develop and improve alongside each other (McClelland and Cameron, 2019). The theory of automaticity posits that the performance of motor and cognitive tasks compete for the same attentional resources. Performance of a new motor task requires strong allocation of cognitive-attentional resources; but with practice resulting in automaticity of behavior, fewer cognitive-attentional resources are needed for successful performance (Floyer-Lea and Matthews, 2004). Thus, if a certain skill is automated, more attentional resources are available for executing cognitive processes (Cameron et al., 2015). Correspondingly, EFs are assumed to no longer be involved in automated motor tasks, making the simultaneous performance of a second EF-demanding task easier (Floyer-Lea and Matthews, 2004). In light of these theories, the development of motor skills and EFs can be seen as multilevel, interactive, and bidirectional (McClelland and Cameron, 2019).

Empirical examination of the relationship between motor skills and EFs is hampered by conceptual challenges such as lack of clarity regarding the structure of motor skills and EFs (Magill and Anderson, 2017; Karr et al., 2018). With regard to motor skills, there is debate as to whether motor performance is based on task specificity or a general motor construct. The *Specificity of Motor Ability Hypothesis* states that motor skills are specific to a particular task and are relatively independent from each other (Magill and Anderson, 2017). In other words, improvement in one motor skill does not ensure improvement in other motor skills. This hypothesis is supported by empirical studies showing low correlations between individual motor skill items in children (Haga et al., 2008; Lorås and Sigmundsson, 2012; Stöckel and Hughes, 2016; Gísladóttir et al., 2019). For example, Haga et al. (2008) found mostly no or non-significantly weak correlations between items of the Movement Assessment Battery for Children in 4-year-old children. The specificity of motor skills is further supported by studies in children showing that practice and experience with one motor skill have limited beneficial effects for the development and learning of other motor skills (Revie and Larkin, 1993). In contrast, the *General Motor Ability Hypothesis* claims the existence of a unitary motor construct underlying a wide range of related motor skills. Hands et al. (2018) suggest that low correlations between motor items do not dismiss a unitary underlying motor construct. For example, studies that have used statistical procedures such as factor analysis have supported an underlying unitary motor construct in children (Ibrahim et al., 2011; Schulz et al., 2011; Okuda et al., 2019). Based on these hypotheses, the current study will focus on both task-specific as well as latent relationships between motor skills and EFs.

**FIGURE 1:**
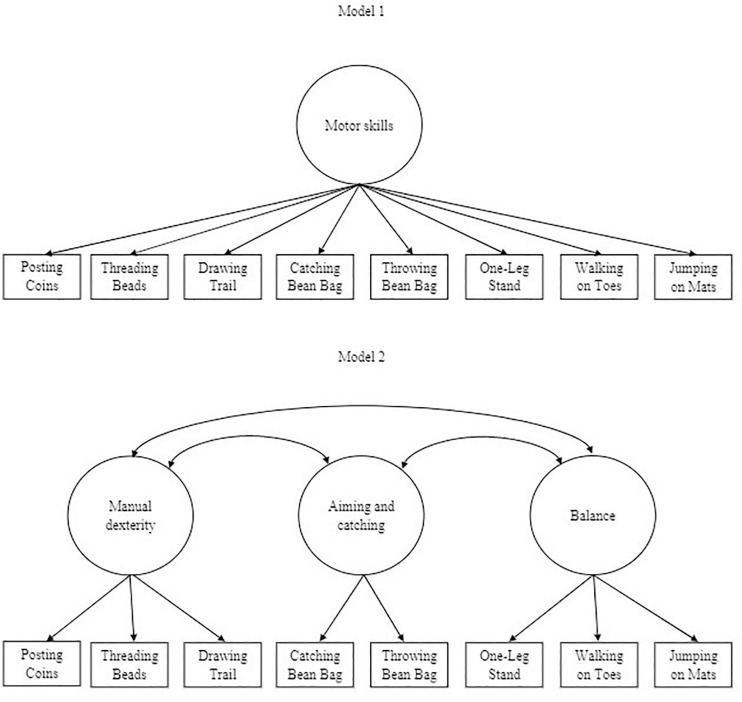
One-factor model of motor skills. Model 1 illustrates latent motor skills as a one-factor structure. Model 2 depicts latent motor skills as a three-factor structure, based on the structure of the Movement Assessment Battery for Children Second Edition (Henderson et al., 2007).

In this study, we will use the Movement Assessment Battery for Children Second Edition (MABC-2, Henderson et al., 2007) to assess motor skills, as this is a comprehensive and widely used instrument for assessing motor proficiency and identifying motor coordination difficulties in children (Blank et al., 2019). Some studies have been conducted with regard to reliability and validity of this instrument, and these studies have shown good-to-excellent test-retest reliability and acceptable-to-good internal consistency (Ellinoudis et al., 2011; Smits-Engelsman et al., 2011). In addition, the MABC-2 discriminates reliably between typically developing children and children having motor coordination difficulties (Ellinoudis et al., 2011). With regard to the original three-factor structure of the MABC-2, mixed findings have been shown. Whereas the original three-factor structure of the MABC-2 has been replicated in preschool children (Psotta and Brom, 2016), other studies were not able to replicate the original three-factor structure but found a one-factor structure instead (Schulz et al., 2011; Okuda et al., 2019). Based on the original structure of the MABC-2 and the outcomes of previous studies regarding its factor structure, the present study takes into account both a one-factor structure and the original three-factor structure of the MABC-2 in examining the latent relationship between motor skills and EFs in the current study (Figure 1).

There is currently no univocal scientific consensus on the definition of EFs, but they are often defined as a set of higher-order cognitive processes that contribute to effortful, purposeful, and problem-solving behavior, with inhibition, working memory, and cognitive flexibility as its core components (Diamond, 2013). The model from Diamond (2013) suggests that the core components are the basis for more complex EFs, such as reasoning, problem solving, and planning, which start to develop at school-age. Studies examining the structure of EFs in preschool children, more precisely the extent to which EF corresponds to a unitary construct or encompasses separable but related components, have shown mixed results (Karr et al., 2018). Several studies concluded EF to be a unitary construct in preschool children (Wiebe et al., 2008, 2011; Shing et al., 2010; Willoughby et al., 2010, 2012; Fuhs and Day, 2011; Masten et al., 2012), while other studies focusing on preschool children identified a two-factor structure with inhibition and working memory/cognitive flexibility as latent components (Miller et al., 2012; Lerner and Lonigan, 2014; Usai et al., 2014; Monette et al., 2015) or a three-factor structure with inhibition, working memory, and cognitive flexibility as latent components (Hughes, 1998; Monette et al., 2011). It should be noted that cognitive flexibility tasks were not always included in the studies that examined the structure of EFs. Studies focusing on preschool children might have excluded it, because it is argued that cognitive flexibility emerges later in development (Müller and Kerns, 2015). There is thus no uniformity in the previous studies about the EF factor structure.

**FIGURE 2:**
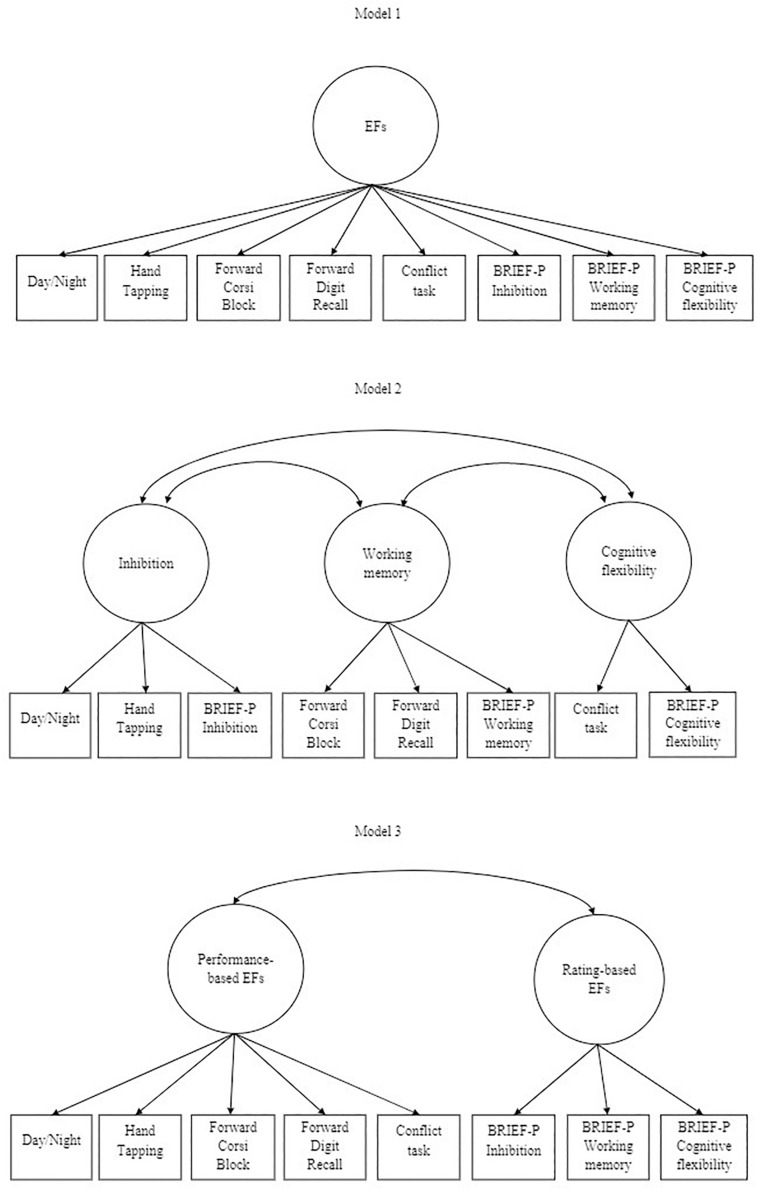
Factor models of EFs. Model 1 illustrates latent EFs as a one-factor structure. Model 2 depicts latent EFs as a three-factor structure. Model 3 illustrates latent EFs as a two-factor structure.

In order to provide a comprehensive examination of children’s EFs, it has been suggested to include both performance-based and rating-based measures (Toplak et al., 2013). Some researchers have suggested that performance-based and rating-based measures assess different aspects of EFs in school-aged children (Toplak et al., 2013), but empirical studies using both types of measures in preschool children have shown significant moderate correlations next to non-significant correlations (Loe et al., 2015; Miranda et al., 2015; Garon et al., 2016). Thus, research up until the present indicates that the extent to which performance-based and rating-based EF measures assess different or similar aspects of EFs in this age range remains unclear. Based on the findings regarding the relationship between performance-based and rating-based EF measures and the different structures of EFs in preschool children, the current study takes three different EF factor structures into account in examining the latent relationship between motor skills and EFs, as displayed in Figure 2: (1) a one-factor structure with performance-based and rating-based EFs, (2) a three-factor structure where inhibition, working memory, and cognitive flexibility were treated as separate latent constructs, and (3) a two-factor structure where performance-based EFs and rating-based EFs were treated as separate latent constructs.

In reviewing the empirical literature on the relationship between motor skills and EFs in preschool children, it has become clear that findings across studies are difficult to compare because of substantial disparities in methodologies. First, previous studies on the relationship between motor skills and EFs in preschool children have mainly focused on a limited set of motor skills and EF domains. For example, some studies have linked only fine motor skills to the three core components of EF (Michel et al., 2011; Roebers et al., 2014; Fang et al., 2017), while other studies have linked only gross motor skills to one or multiple EF domains (Heibel-Witte, 2016; Cook et al., 2019). These studies have shown inconsistent results regarding the relationship between motor and EF domains. For example, Fang et al. (2017) found significant weak relationships between fine motor skills, and inhibition and cognitive flexibility in children aged 4 to 6 years, but not between fine motor skills and working memory. Contrary to the results of Fang et al. (2017), Michel et al. (2011) found no significant relationships between fine motor skills and inhibition, working memory, and cognitive flexibility in typically developing children aged 5 to 7 years and a significant moderate relationship between fine motor skills and working memory in 5- to 7-year-old children with motor coordination difficulties, but not between fine motor skills, and inhibition and cognitive flexibility. Previous studies that used multiple tasks for measuring an EF domain have also reported inconsistent results between motor skills on the one hand and the tasks targeting the same EF domain on the other hand (Livesey et al., 2006; Lehmann et al., 2014). For example, Livesey et al. (2006) examined the relationship between motor skills and different inhibition tasks in 5- to 6-year-old children. Significant moderate-to-strong relationships were found the motor component scores of the MABC-2 and the ‘Day/Night,’ while non-significant relationships were found between the motor component scores of the MABC-2 and the Stop-Signal task. The inconsistent results suggest that the relationship between motor skills and EFs in preschool children is task-specific and emphasize the need for a comprehensive examination of their relationship.

This task-specificity of the relationship between motor skills and EFs has been suggested in previous studies (Roebers and Kauer, 2009; Michel et al., 2011; Stöckel and Hughes, 2016). If the relationship between motor skills and EFs is task-specific, it can be expected that interventions targeting specific motor skills also influence specific EFs, yet not all EFs. Inversely, because of the bidirectional relationship between motor skills and EFs (e.g., Roebers et al., 2014), interventions targeting specific EFs are expected to also influence specific motor skills, yet not all motor skills. Based on this assumption, interventions should focus on specific motor skills and EFs, and professionals should choose interventions carefully by selecting interventions that target the specific skills of interest. Therefore, the inclusion of a comprehensive variety of motor skills and EFs is important in providing a more comprehensive view of how motor skills and EFs are related during the preschool period.

Another methodological disparity is that previous studies in preschool children have examined the relationship between motor skills and EFs on exclusively one level (i.e., on an item-level or on a construct-level). Most studies have linked specific component scores of a motor test (on a construct-level) to specific EF task scores (on an item-level) (e.g., Livesey et al., 2006; Michel et al., 2011; Lehmann et al., 2014; Alesi et al., 2019). These studies showed widely varying relationships, even within a specific study, from non-significant to significantly strong. Three other studies examined latent relationships between motor skills and EFs by linking latent components of motor skills (in these cases: fine motor and/or gross motor skills) to a unitary latent factor of EF (Roebers et al., 2014; Oberer et al., 2017; Maurer and Roebers, 2019). These studies found moderate-to-strong relationships. Furthermore, some studies examined task-specific relationships between motor skills and EFs and reported relationships varying from moderate to none; however, most of the relationships found were weak relationships (Roebers et al., 2014; Fang et al., 2017; Oberer et al., 2017; Maurer and Roebers, 2019). In conclusion, there seems to be no clear pattern in the findings from the use of specific items, latent constructs, or a combination of the two. In order to get more insight into the multi-level nature of the relationship between motor skills and EFs, it is important to examine both task-specific and latent relationships in one study. Such a comprehensive examination will provide more fine-grained information on what and how components of motor skills and EFs are related.

In summary, the multi-level nature of the relationship between motor skills and EFs in preschool children is still not well-understood and further research is warranted. Therefore, the aim of the current study was to get a more fine-grained understanding of the multi-level nature of the relationship between motor skills and EFs in preschool children by examining their task-specific and latent relationships, including a range of motor skills (i.e., manual dexterity, aiming and catching, and balance skills) and EFs (i.e., performance-based and rating-based inhibition, working memory, and cognitive flexibility). This study took an exploratory approach with regard to the relationship between motor skills and EFs, because of 1) the mixed findings of empirical studies focusing on the relationship between motor skills and EFs in preschool children and 2) the absence of studies examining both task-specific and latent relationships between motor skills and EFs.

## Materials and Methods

### Participants

The current study sample was part of a larger research project ‘MELLE’ (Motor skills, Executive functions, Language, and LEarning outcomes in preschool children; see also Houwen et al., 2019) in which 3- to 5-year old children are followed regarding their motor skills, EFs, and language abilities. From April 2016 to October 2019 a sample of 207 typically developing children was recruited from kindergartens, preschools, primary schools, and day-care centers, and via social media, flyers and posters distributed at supermarkets, stores, and playgrounds. Inclusion criteria were: (a) age between 36 and 72 months, (b) no signs of a medical condition (e.g., heart disease), neurological disorder (e.g., cerebral palsy), or intellectual or physical disability (e.g., club foot), (c) normal hearing and normal or corrected to normal vision, (d) being able to follow the test instructions, and (e) having parents/caretakers who have sufficient proficiency in written Dutch to be able to complete the questionnaires.

Nine children (4.4%) were removed from the original sample because of more than 50 percent missing values due to refusal, lack of concentration, or motivation. All of these nine children were 3- or 4-year old children with the majority being 4 years of age (*n* = 6; 66.7%). Most of these excluded children were girls (*n* = 6; 66.7%). Therefore, the final sample consisted of 198 children (102 boys; 51.5%), aged 36 to 71 months old (*M* = 50.9 months, *SD* = 10.1 months). The sample consisted of 83 3-year olds (42 boys; 50.6%), 63 4-year olds (36 boys; 57.1%), and 52 5-year olds (24 boys; 46.2%).

### Instruments

#### Motor Skills

Motor skills were assessed with age band 1 of the Movement Assessment Battery for Children Second Edition – Dutch version (MABC-2; Henderson et al., 2010). It consists of eight tasks divided over three subscales: (1) manual dexterity, which consists of the items ‘Posting Coins,’ ‘Threading Beads,’ and ‘Drawing Trail,’ (2) aiming and catching, which includes the items ‘Catching a Bean Bag’ and ‘Throwing a Bean Bag,’ and (3) balance, which consists of the items ‘One-Leg Stand,’ ‘Walking on Toes,’ and ‘Jumping on Mats.’ Raw items scores were transformed to age-based item standard scores (range = 1–19, *M* = 10, *SD* = 3). Age band 1 of the MABC-2 has been found to be a reliable and valid measure to assess motor skills in preschool children (Ellinoudis et al., 2011; Smits-Engelsman et al., 2011; Psotta and Brom, 2016).

#### Performance-Based EFs

Five performance-based EF measures were used for inhibition, two for working memory, and one for cognitive flexibility. The EF tasks were age adequate and required either a motor or verbal response. The raw task scores of all performance-based EF measures were converted into z-scores per age group.

#### Inhibition

The ‘Day/Night’ (Gerstadt et al., 1994) is a verbal inhibition task. Children were shown black cards with a moon and stars and white cards with a sun. They were requested to say “day” to the moon card and “night” to the sun card. The task started with two practice cards followed by 16 test cards. The test cards were presented in the following order: moon card (m), sun card (s), s, m, s, m, m, s, s, m, s, m, m, s, m, s. Similar terms for day and night were scored as correct (e.g., “light” or “sun” instead of day). The amount of correct responses was scored (0-16). Studies have shown good internal consistency (Chasiotis et al., 2006; Rhoades et al., 2009) and test-retest reliability (Thorell and Wåhlstedt, 2006) in preschool children.

The ‘Hand Tapping’ (Diamond and Taylor, 1996) is a fine motor inhibition task. Children were asked to tap once when the tester tapped twice and to tap twice when the tester tapped once. The task consisted of two practice trials and 16 test trials. The series of the tester’s tap was as follows: 1, 2, 2, 1, 2, 2, 1, 1, 1, 2, 1, 2, 2, 1, 1, 2. The number of correct responses (0-16) was scored. Studies have reported good internal consistency (Blair and Razza, 2007; Bierman et al., 2008; Rhoades et al., 2009).

#### Working Memory

The ‘Forward Corsi Block’ (Pickering et al., 1998) is a visuo-spatial working memory task. Children were asked to reproduce the same sequence of blocks tapped by the tester. Number sequences increased from two to six blocks, with three trials per sequence length. The test was terminated after three incorrect responses within one sequence length. The total score was the number of correct responses (0-15). The ‘Forward Corsi Block’ showed good test-retest reliability in preschool children (Alloway et al., 2006, 2009).

The ‘Forward Digit Recall’ (Gathercole and Pickering, 2000) is a verbal working memory task. Children were requested to recall the numbers said by the tester in the same order. Number sequences increased from two to seven digits, with three trials per sequence length. The test was terminated after three incorrect responses within one sequence. The total score was the number of correct responses (0-18). The test-retest reliability of the ‘Forward Digit Recall’ has been reported to be acceptable to good in preschool children (Alloway et al., 2006; Müller et al., 2012).

#### Cognitive Flexibility

We used a modified version of the ‘Conflict Task’ (Beck et al., 2011) for measuring cognitive flexibility using fine motor demands, which is adapted from the standard Dimensional Change Card Sorting task (Zelazo, 2006). The children were presented with two recipe boxes with slots cut in the top. A yellow target card with an airplane was attached to the front of one box. A red target card with a truck was attached to the front of the other box. The task consisted of two levels. In the first level, children were presented yellow cards with trucks and red cards with airplanes. They were asked to sort the cards according to the shape for six trials and then to sort the cards according to the color for six trials. In the second level, some of the cards contained a black border around the card and some did not. Children were asked to sort by color if the card had a black border around it and by shape if the card did not have a black border around it. This level consisted of a practice phase, in which the children practiced four cards, two with borders and two without. In line with Beck et al. (2011), the children were presented six cards with a border (B) and six cards without a border (NB) in the following order: B, NB, B, B, NB, NB, B, B, NB, NB, B, NB. The total score was the number of correct responses of both levels (0-18). Both levels of the ‘Conflict Task’ have shown good test-retest reliability (Beck et al., 2011).

#### Rating-Based EFs

The Dutch version of the Behavior Rating Inventory of Executive Function – Preschool version (BRIEF-P; Van der Heijden et al., 2013) is a standardized questionnaire consisting of 63 items that measure everyday EF in the home environment of children aged 35 to 71 months old. Parents were requested to rate how often their child exhibited various behaviors related to EF in the past 6 months on a three-point scale (1 = never, 2 = sometimes, 3 = often). Corresponding to the performance-based EF component measures, only the subscales Inhibition (16 items; e.g., “Is impulsive”), Working Memory (17 items; e.g., “Has trouble finishing tasks”), and Cognitive Flexibility (10 items; e.g., “Is upset by change in plans and routines”) were included in the current study. Age- and gender-corrected T-scores (*M* = 50, *SD* = 10) were calculated for the three subscales in which higher scores are indicative of poorer EF. The T-scores were reversed in order to keep the interpretation of all tests in the same direction, namely higher scores reflecting better EFs. The Dutch version of the BRIEF–P showed sufficient to high internal consistency, test–retest reliability, interrater reliability, and construct validity (Van der Heijden et al., 2013).

### Procedure

The study protocol was approved by the Ethics Review Committee of the Department of Pedagogical and Educational Sciences, Faculty of Behavioural and Social Sciences, University of Groningen. All parents gave written informed consent in accordance with the Declaration of Helsinki. The data were collected by graduate students in Pedagogical and Educational Sciences, Psychology, and Human Movement Sciences. Before they were allowed to collect any data, they had to follow and pass an extensive training. As part of the training, they read test manuals and followed two training sessions in which they practiced administering the tests on each other. Furthermore, they performed two video-taped practice assessments with a preschool child on which they were provided individual feedback.

Data collection consisted of two home sessions, each lasting 90 to 120 minutes, in which the children performed several motor, cognitive, and language tests as part of the MELLE study. The assessments were videotaped for scoring purposes, and to allow for later review of the data and fidelity in following testing procedures. Children were encouraged with stickers after every task. When necessary, breaks were used to maintain attention and motivation. After each assessment, children received a small gift and a diploma. Parents filled out questionnaires on their child’s development, behavior, and daily environment. Parents received a report with the test results of their child. To ensure confidentiality, data were entered and stored using a personalized study identifier.

### Analysis

Missing value analysis was conducted using SPSS Version 25 (IBM Corporation, 2017). Little’s MCAR test was used to evaluate the missing at random pattern of missing values. Assumptions of normality, linearity, and homoscedasticity were examined with boxplots, histograms, and scatterplots. The BRIEF-P subscale scores were transformed by multiplying the scores with -1 in order to be consistent with the motor and EF task scores (where higher scores reflect better functioning). Relationships between specific items of motor skills and EFs were examined with zero-order Pearson correlation analysis.

In order to be able to merge the data of 3-, 4-, and 5-year olds, we tested the invariance of the correlation matrices separately for the motor and the EF scores across age groups by means of multi-group invariance testing in LISREL 8.8 (Jöreskog and Sörbom, 2006). As a main indication of model fit, the ratio of χ^2^ to the degrees of freedom (χ^2^/*df*) was used. In contrast to χ^2^ or the *p*-statistic, the χ^2^/*df*-measure is less sensitive to group sizes and departures from normality (Marsh and Hocevar, 1985; Byrne, 1989). According to Byrne (1989), a χ^2^/*df*-ratio equal to or below 2 can be considered a good fit. The comparative fit index (CFI) (Bentler, 1990) was also examined as an additional indication of model fit. This index also reflects the model fit relatively well at all sample sizes. All further analyses were performed in MPlus Version 8.3 (Muthén and Muthén, 2019).

**FIGURE 3:**
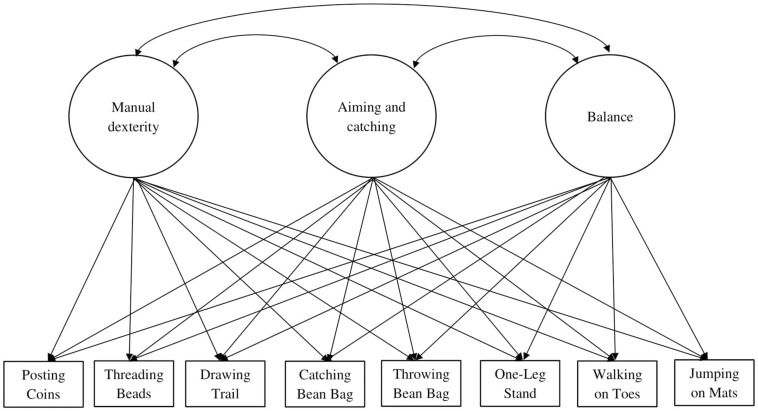
Exploratory structural equation model of the three-factor model of motor skills.

To test the fit between the latent structure of motor skills and EFs in 3- to 5-year-old children, we started with performing confirmatory factor analysis (CFA) as this analysis is the most parsimonious (Marsh et al., 2014). Modification indices were analyzed to examine whether and how the model fit could be improved with the addition of co-variances. Hence, from a two-factor model onward, we performed exploratory structural equation modeling (ESEM) in addition to CFA, because this analysis is less restrictive to fit the observed data than CFA and it allows cross-loadings between factors (Marsh et al., 2014). An example of an ESEM is shown in Figure 3. As suggested by Marsh et al. (2013), the ESEM factor structure was used in further analysis when the ESEM results fitted the data better than the corresponding CFA model results, with the absence of inflated fit indices. Otherwise, the CFA factor structure was used, on the basis of parsimony. Regarding ESEM, Geomin, an oblique rotation method, was used to establish the optimum pattern of item loadings. The factor structures that were tested for motor skills were (1) a one-factor structure, and (2) a three-factor structure consisting of fine motor skills, ball skills, and balance. The factor structures that were tested for EF were (1) a one-factor structure with performance-based and rating-based EFs, (2) a three-factor structure where inhibition, working memory, and cognitive flexibility were treated as separate latent constructs, and (3) a two-factor structure where performance-based EFs and rating-based EFs were treated as separate latent constructs. The model fit was evaluated using several model fit measures. Criteria for good model fit were low values of χ^2^ (with a corresponding non-significant *p*-value, indicative of a non-significant discrepancy between the data and the imposed factor structure), Root Mean Square Error of Approximation (RMSEA) and Standardized Root Mean Square Residual (SRMSR) < 0.08, and CFI and Tucker-Lewis Fit Index (TLI) > 0.90. It should be noted that, although these are the best model fit measures available for ESEM, there is incomplete evidence to confirm that these model fit measures are suitable to be used in ESEM studies (Marsh et al., 2009).

Next, the motor model and EF model that showed a good fit to the data were related to each other using SEM in order to evaluate relationships between latent variables of motor skills and EF. To evaluate the goodness-of-fit of the structural equation model, χ^2^, RMSEA, CFI, TLI, and SRMSR were used.

## Results

### Preliminary Analyses

Descriptive statistics are provided in Table 1. The rate of missingness varied from 0% (age and gender) to 22.2% (‘Day/Night’) (see Table 1 for the amount of available data per variable). Little’s MCAR test indicated the missing values (6.6%) were not missing completely at random (Little’s MCAR test: χ^2^ (626) = 719.719, *p* = 0.005). Taking a more detailed look at the missing values, the pattern of missingness appeared to be dependent on age and gender. Three-year-old children had more missing values than four- and five-year-old children. Additionally, boys had more missing values than girls. The missingness appeared to be related to observed variables (i.e., age and gender), which supports the use of multiple imputation under missing at random conditions (Graham, 2009). Analyses of scatterplots and boxplots showed neither significant outliers, nor violations of linearity and homoscedasticity assumptions. Histograms revealed small deviations from normality for the ‘Drawing Trail,’ ‘Jumping on Mats,’ ‘Day/Night,’ ‘Hand Tapping,’ and the BRIEF-P subscales.

**TABLE 1:** Descriptive statistics for original motor and EF scores.

	Original			
	
	*n*	*M*	*SD*	*Range*
Posting Coins	193	10.57	2.46	2–14
Treading Beads	194	10.13	2.53	1–16
Drawing Trail	197	9.25	2.49	1–15
Catching a Bean Bag	194	9.41	2.90	1–16
Throwing a Bean Bag	197	10.16	3.13	2–19
One-leg Stand	190	8.38	2.34	3–17
Walking on Toes	186	9.61	3.07	2–15
Jumping on Mats	195	9.53	3.14	1–12
BRIEF-P Inhibition	193	–50.80	10.09	−88–−34
BRIEF-P Working Memory	193	–51.55	10.22	−84–−36
BRIEF-P Cognitive Flexibility	193	–51.66	10.74	−82–−37
Day/Night	154	0.00	0.99	−3.84–1.45
Hand Tapping	156	0.00	0.99	−4.85–1.50
Forward Corsi Block	159	0.00	0.99	−2.57–3.54
Forward Digit Recall	166	0.00	0.99	−2.37–3.11
Conflict task	174	0.00	0.99	−3.05–1.87

*BRIEF-P: Behavior Rating Inventory of Executive Functioning - Preschool version.*

The multi-group invariance assumption of the correlation matrices across the 3-, 4-, and 5-year olds on the eight motor items was not rejected by the data (χ^2^ = 51.25, *df* = 72, χ^2^/*df* = 0.71, *p* = 0.97, *p*-value for test of close fit, RMSEA = 0.99, CFI = 1.00). In addition, inspection of modification indices also did not reject the multi-group invariance assumption. The multi-group assumption of invariance of the correlation matrices across the 3-, 4- and 5-year olds on the eight EF items was not rejected by the data (χ^2^ = 50.05, *df* = 72, χ^2^/*df* = 0.69, *p* = 0.98, *p*-value for test of close fit, RMSEA = 0.99, CFI = 1.00). Inspection of the modification indices did not point to violations of the invariance either. Hence, the aggregation of the correlational motor skills and EF data across age groups is highly defendable.

### Relationship Between Specific Motor and EF Items

To account for potential bias resulting from missing data and to increase statistical power in correlation analysis, multiple imputation with full conditional specification was performed in SPSS 25 (IBM Corporation, 2017). Age and gender were used as predictors and the motor skill and EF variables were used as predictors and variables to be imputed. Twenty multiple imputed data sets were created, as this amount of imputed data sets leads to a preventable power fall off of less than 1% (Graham et al., 2007). Cohen’s *d* analysis demonstrated that the original and pooled means of the motor and EF scores were similar for the whole sample (*d* = 0.00 to *d* = 0.07; Cohen, 1988). Pearson correlation analysis was performed on each imputed data set individually and the results were pooled by making use of SPSS tabular output by default.

Data for some variables were not normally distributed; however, with a sample size of 198, the central limit theorem suggests that parametric tests (Pearson correlation) would still be sufficiently robust to avoid deviations from normality. Table 2 provides an overview of the zero-order correlations between the specific motor and EF items. Statistically significant, albeit weak, positive correlations (*r* = 0.15 to *r* = 0.22) were found between all manual dexterity tasks and ‘Hand Tapping.’ Furthermore, the ‘Drawing Trail’ and ‘Jumping on Mats’ task correlated significantly, but weakly, positively with the ‘Forward Corsi Block’ (*r* = 0.17 to *r* = 0.23). There were no significant correlations between the motor tasks and the BRIEF-P subscales.

**TABLE 2:** Bivariate correlations between specific motor and EF items.

								Conflict
	BRINH	BRWM	BRCF	DN	HT	Corsi	DR	task
MD1	0.00	0.03	0.05	0.02	0.22^∗∗^	0.08	0.07	0.09
MD2	0.03	0.07	0.04	0.06	0.16^∗^	0.08	0.00	0.13
MD3	–0.01	0.02	–0.10	0.12	0.15^∗^	0.23^∗∗^	0.09	0.12
AC4	0.13	0.03	0.09	0.02	–0.01	0.05	–0.05	0.08
AC5	0.03	–0.03	–0.05	–0.02	–0.10	–0.07	–0.04	–0.07
BA6	0.11	0.13	0.12	0.04	0.06	0.14	0.05	0.02
BA7	0.00	–0.01	0.08	0.11	0.03	0.01	0.08	0.15
BA8	0.13	0.09	0.04	0.06	0.02	0.17^∗^	–0.11	0.05

*p < 0.05; **p < 0.01 (two-tailed). MD1: Posting Coins; MD2: Threading Beads; MD3: Drawing Trail; AC4: Catching a Bean Bag; AC5: Throwing a Bean Bag; BA6: One-leg Stand; BA7: Walking on Toes; BA8: Jumping on Mats; BRINH: Behavior Rating Inventory of Executive Function Preschool Version (BRIEF-P) Inhibition subscale; BRWM: BRIEF-P Working Memory subscale; BRCF: BRIEF-P Cognitive Flexibility subscale; DN: Day/Night; HT: Hand Tapping; Corsi: Forward Corsi Block; DR: Forward Digit Recall.

### Factor Structure of Motor Skills

Because of the complexity of the analyses in MPlus, we accounted for deviations from normality in combination with missing values using robust maximum likelihood estimation (MLR) (Savalei, 2010). Based on the empirical evidence in favor of two models (as described in the introduction), we considered two models, displayed in Figure 1. Regarding CFA, Table 3 provides an overview of the model fit statistics of the different factor structures. The first model we tested using CFA was a one-factor structure with all MABC-2 items (Model 1a). Model fit indices were found to be poor. Inspection of modification indices suggested that the model fit could be improved by allowing ‘Posting Coins’ and ‘Threading Beads,’ and ‘Catching a Bean Bag’ and ‘Throwing a Bean Bag’ to covariate. These modifications were justified from a theoretical point of view, since ‘Posting Coins’ and ‘Threading Beads’ are both speed items (Henderson et al., 2007). ‘Catching a Bean Bag’ and ‘Throwing a Bean Bag’ are both items that require control of fast-moving objects (Utley, 2019) and may be highly dependent on practice and experience (Henderson et al., 2007). Furthermore, these tasks correlated moderately with each other (*r* = 0.42 and *r* = 0.30, respectively, both *p* < 0.01). After adding these co-variances to the model, the one-factor model (Model 1b) revealed a good model fit (see Table 3). All factor loadings (λ) were statistically significant (*p* < 0.01; see Table 4), indicating they were good indicators of the latent factor. The second model (Model 2) we tested using CFA was the three-factor structure of the MABC-2 with ‘Posting Coins,’ ‘Threading Beads,’ and ‘Drawing Trail’ representing manual dexterity; ‘Catching a bean bag’ and ‘Throwing a Bean Bag’ representing aiming and catching skills; and ‘One-leg Stand,’ ‘Walking on Toes,’ and ‘Jumping on Mats’ representing balance. The model fit indices were found to be poor. Inspection of modification indices showed that the three-factor model could not be improved by allowing tasks to covariate. Using ESEM, convergence of the three-factor model (Model 2) could not be achieved because of a negative residual variance for ‘Threading Beads.’ In conclusion, the results indicated the existence of a one-factor structure with a latent general motor factor in this sample of 3- to 5-year-old children.

**TABLE 3:** Model fit statistics of the different factor structures of motor skills examined with CFA.

	χ^2^ (*p*)	RMSEA	CFI	TLI	SRMR
Model 1a	53.62 (0.00)	0.09	0.80	0.72	0.06
Model 1b	30.61 (0.03)	0.06	0.92	0.88	0.05
Model 2	35.16 (0.01)	0.07	0.89	0.82	0.05

*CFA: confirmatory factor analysis; Model 1a: one-factor structure; Model 1b: one-factor structure with modifications; Model 2: three-factor structure; RMSEA: Root Mean Square Error of Approximation; CFI: Comparative Fit Index; TLI: Tucker-Lewis Fit Index; SRMR: Standardized Root Mean Square Residual.*

**TABLE 4:** Factor loadings and residual errors of the motor and EF models.

One-factor motor model				Two-factor EF model			
	
Latent factor	Items	Λ	ε	Latent factor	Items	λ	ε
General motor skills	Posting Coins	0.44	0.81	Performance-based EFs	Day/Night	0.31	0.90
	Threading Beads	0.53	0.72		Hand Tapping	0.26	0.93
	Drawing Trail	0.40	0.84		Corsi Block	0.66	0.57
	Catch a Bean Bag	0.44	0.81		Digit Recall	0.59	0.65
	Throwing a Bean Bag	0.22	0.95		Conflict task	0.37	0.86
	One-leg Stand	0.54	0.71	Rating-based EFs	BRIEF-P Inhibition	0.88	0.23
	Walking on Toes	0.44	0.81		BRIEF-P Working Memory	0.81	0.35
	Jumping on Mats	0.38	0.86		BRIEF-P Cognitive Flexibility	0.47	0.78

*BRIEF-P: Behavior Rating Inventory of Executive Function Preschool Version.*

### Factor Structure of EFs

As described in the introduction, we considered three models, displayed in Figure 2. Regarding CFA, Table 5 provides an overview of the model fit statistics of the different factor models. The first model we tested using CFA was a one-factor structure with all performance-based and rating-based EF items (Model 1a). Model fit indices were found to be poor. Inspection of modification indices suggested that the model could be improved by allowing the ‘Forward Digit Recall’ and ‘Forward Corsi Block’ to covariate. These modifications were justified from a theoretical point of view, because these EF items both assess the ability of remembering and reproducing sequences of information (Pickering et al., 1998; Gathercole and Pickering, 2000) and correlated moderately with each other (*r* = 0.39, *p* < 0.01). After setting these co-variances to be free, the one-factor model (Model 1b) still showed poor model fit. The second model (Model 2) we tested using CFA was a three-factor structure with the ‘Day/Night,’ ‘Hand Tapping,’ and BRIEF-P Inhibition subscale representing inhibition; the ‘Forward Corsi Block,’ ‘Forward Digit Recall,’ and BRIEF-P Working Memory subscale representing working memory; and the ‘Conflict Task’ and BRIEF-P Cognitive Flexibility subscale. The model fit indices were found to be poor. Inspection of modification indices showed the three-factor model could not be improved by allowing tasks to covariate. Using ESEM, convergence of the three-factor model (Model 2) could not be achieved because of a negative residual variance for ‘Day/Night.’ The third model (Model 3) we tested using CFA was a two-factor model with the EF tasks representing performance-based EFs and the BRIEF-P subscales representing rating-based EFs. The model fit indices were found to be good. All factor loadings (λ) were statistically significant (*p* < 0.01; see Table 4), indicating that all EF items were good and unique indicators of the latent factors. The two-factor model (Model 3) was also examined using ESEM. Model fit indices were RMSEA = 0.00, CFI = 1.00, TLI = 1.02, SRMR = 0.03. In conclusion, CFA and ESEM supported the existence of a two-factor structure with a performance-based EF and a rating-based EF factor in this sample of 3- to 5-year-old children. The CFA model of the two-factor structure was used in further analysis, because of its parsimonious character and the unrealistically high model fit indices found with ESEM.

**TABLE 5:** Model fit statistics of the different factor structures of EFs examined with CFA.

	χ^2^ (*p*)	RMSEA	CFI	TLI	SRMR
Model 1a	64.77 (0.00)	0.11	0.80	0.72	0.10
Model 1b	45.24 (0.00)	0.08	0.88	0.82	0.09
Model 2	55.07 (0.00)	0.11	0.83	0.72	0.10
Model 3	20.80 (0.35)	0.02	0.99	0.99	0.05

*Model 1: one-factor structure; Model 1b: one-factor structure with modifications; Model 2: three-factor structure; Model 3: two-factor structure; RMSEA: Root Mean Square Error of Approximation; CFI: Comparative Fit Index; TLI: Tucker-Lewis Fit Index; SRMR: Standardized Root Mean Square Residual.*

### Relationship Between Models of Motor Skills and EFs

**FIGURE 4:**
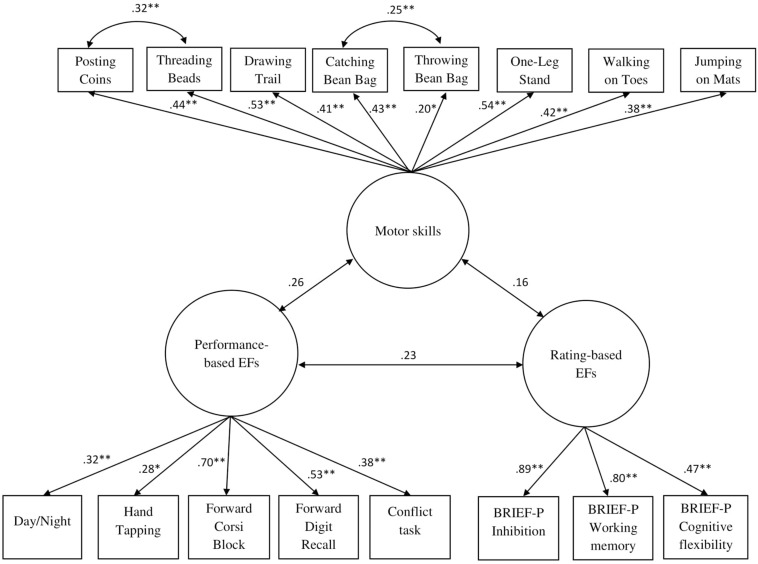
Structural equation model of the relationships between motor skills and EFs. *p < 0.05; **p < 0.01.

The one-factor motor model (Model 1b) was related to the two-factor EF model (Model 3) by means of SEM (Figure 4). The model fit the data well (χ^2^ = 117.87, RMSEA = 0.03, CFI = 0.95, TLI = 0.94, SRMR = 0.06). However, the latent factor of motor skills correlated non-significantly and weakly with the latent factor of performance-based EFs (*r* = 0.26, *p* = 0.08) and of rating-based EFs (*r* = 0.16, *p* = 0.14).

## Discussion

### Main Findings

The current study investigated the task-specific and latent relationship between motor skills and EFs in preschool children. The correlations between specific motor and EF items showed significant albeit weak relationships between both domains, i.e., between all manual dexterity tasks and ‘Hand Tapping’; and between the ‘Drawing Trail,’ ‘One-Leg Stand,’ and ‘Jumping on Mats’ tasks and the ‘Forward Corsi Block.’ There were no significant correlations between any of the specific motor items and the rating-based EF subscale scores. The SEM model revealed non-significant weak relationships between a general motor factor (as a unitary latent construct) and the latent constructs of performance-based EFs and rating-based EFs. There were no clear differences in results neither between the relationship between specific items of motor skills and EFs, nor between the unitary latent motor construct and latent components of EFs.

In line with the results concerning the task-specific relationships, Maurer and Roebers (2019) found significant weak relationships between the fine motor items of the MABC-2 and an inhibition task; and weak relationships between the ‘Drawing Trail’ and, the balance items of the MABC-2, and a working memory task in a sample of 5- to 6-year-old children. In contrast to our findings on an item-level, Maurer and Roebers (2019) obtained significant weak relationships between fine motor skills and a cognitive flexibility task. Oberer et al. (2017) reported more significant and stronger relationships between motor skills and EF tasks in a sample of 5- to 7-year-old children than the current study. Additionally, the results concerning the latent relationship differed from previous studies (Oberer et al., 2017; Maurer and Roebers, 2019). Oberer et al. (2017) showed strong relationships between gross motor skills and EF and Maurer and Roebers (2019) discovered moderate-to-strong relationships between these latent constructs. In addition, both of these studies showed strong relationships between a latent fine motor skills construct and a latent EF construct. The partly different results might be explained by the younger sample in our study compared to the samples of Maurer and Roebers (2019) and Oberer et al. (2017). Early development is characterized by non-linearity: Increases in performance in one developmental domain can be accompanied by decreases in performance in other developmental domains because the child has to divert energy toward the emerging skill at the expense of other areas (Ben-Sasson and Gill, 2014). However, stability of development seems to increase with age (Schneider et al., 2014). Relationships between motor skills and EFs may therefore become stronger as a function of age. In addition, due to discontinuity in (early) development, relationships between motor skills and EFs may be weaker cross-sectionally compared to longitudinally.

Another explanation for the weak relationships between motor skills and EFs in young children may be found in the role child and environmental factors may play in both domains and their relationship. In light of embodied cognition theories, the relationship between motor skills and EFs is shaped by features of the physical body and grounded in the unique experiences within the environment (Adolph and Hoch, 2019). In this context, it could imply that relationships between motor skills and EFs differ per specific subgroups of children. For example, previous studies have demonstrated that gender, attention, ADHD symptomatology, and SES confounded the relationship between motor skills and EFs (Wassenberg et al., 2005; Piek et al., 2008; Houwen et al., 2017). In addition, several studies have mentioned the possible effect of moderators, such as gender, non-verbal intelligence, reaction time, visual perception, and fitness (Aadland et al., 2017; Michel et al., 2018). The potential role of child characteristics and environmental factors as confounding variables and moderators thus need to be taken into account when examining the relationship between motor skills and EFs.

The inconsistent results found in empirical studies (Livesey et al., 2006; Michel et al., 2011; Lehmann et al., 2014; Roebers et al., 2014; Stöckel and Hughes, 2016; Fang et al., 2017; Houwen et al., 2017; Oberer et al., 2017; Alesi et al., 2019; Cook et al., 2019; Maurer and Roebers, 2019), including the current study, may be partially explained by the use of different motor and EF measures. Measurement selection is important when latent relationships are examined, because the common variance across multiple measures captured by latent variables may include measurement error resulting from unintendedly measured common additional processes, such as attention and language comprehension (Friedman and Miyake, 2017). Miller et al. (2012) showed that the structure of EF examined with CFA was influenced by measurement selection in a sample of preschool children. Different factor structures, as a result of different selection of measures, may, subsequently, lead to different latent relationships. In addition, the selection of measures may have influenced the results regarding the task-specific relationships. Measurement selection is a challenge for researchers, especially the selection of EF tasks, because numerous performance-based EF measures have been developed for use in preschool children (Ackerman and Friedman-Krauss, 2017). However, many of these measures have not been thoroughly evaluated for psychometric properties (Willoughby and Blair, 2016). The current study included performance-based EF measures that have shown good internal consistency and/or test-retest reliability in preschool children indicating good psychometric properties (Alloway et al., 2006, 2009; Chasiotis et al., 2006; Thorell and Wåhlstedt, 2006; Blair and Razza, 2007; Bierman et al., 2008; Rhoades et al., 2009; Beck et al., 2011; Müller et al., 2012).

### Limitations

There are some limitations that should be taken into consideration when interpreting the results. First, age and gender were not included in the analysis of the factorial structures and relationships between both domains. Although multi-group invariance testing demonstrated that the correlational data of the age groups could be aggregated, age and gender may have an effect on the results. For example, the factor structure might differ per age group and gender with the consequence that the analyses should be performed per age group and gender, and may result in differential relationships. Unfortunately, although there are no explicit guidelines for the minimum sample size required to examine measurement invariance in factor structure, research into the required minimum sample size for CFA implies that the sample size of the current study was too small to evaluate measurement invariance of the factor structures of motor skills and EFs (Kyriazos, 2018). Thus, we could not examine the potential influence of age and gender on the relationship between both domains. Second, the data contained some missing values (6.6%) which reduced the statistical power and may have led to biased estimates (Kang, 2013). In our study, the missingness was related to observed variables (namely age and gender), which supports the use of multiple imputation under missing at random conditions (Graham, 2009). We attempted, however, to reduce the impact of missing data by using multiple imputation for the correlation analyses and robust maximum likelihood estimation for the CFAs, ESEMs, and SEM. Third, convergence could not be achieved by ESEM for the three-factor model of motor skills and the three-factor model of EFs. The failure of convergence was probably due to an issue regarding the item ‘Threading Beads’ (regarding the motor model) and the ‘Day/Night’ task (regarding the EF model). The failure of convergence might have been caused by measurement errors. The speed element of the item ‘Threading Beads’ might not have been understood well by the preschool children as observed regularly by the test administrators in this study. Regarding the ‘Day/Night’ task, many preschool children have difficulty remembering the rules of this task (Diamond et al., 2002). Fourth, the weak relationships found using SEM were non-significant. The sample size may have been too small to show significant relationships using SEM. *P*-values are highly dependent on sample size and should therefore be interpreted with caution (Cohen, 1990; Cumming, 2014).

### Future Directions and Implications

The fact that only weak relationships were discovered in the current study suggests that motor skills and EFs may be distinct developmental domains at preschool age. In addition, the findings suggest that it may be important that children with both motor and EF difficulties receive intervention targeted at both developmental domains. Intervention studies, however, showed that interventions targeting motor skills had positive effects on EFs in young children (Zoghi et al., 2016; Mulvey et al., 2018). Clearly, more research is required to gain more insight into whether interventions focused on only one developmental domain, such as motor skills, result in sufficient improvement in both developmental domains, or whether interventions focused on both motor skills and EFs are required to be effective in young children. Furthermore, future longitudinal research is needed to explore whether relationships between motor skills and EFs in young children exist over time. Additionally, it would be worth examining the role of potential confounding and moderating factors in the relationship between motor skills and EFs, such as gender, attention, and fitness (Wassenberg et al., 2005; Aadland et al., 2017; Houwen et al., 2017).

The current study’s findings did not confirm the three dimensional structure of the MABC-2 proposed by Henderson et al. (2007). Instead, our study indicated a one-factor structure of motor skills. Although some studies supported the three-factor structure of the MABC-2 in preschool children (Ellinoudis et al., 2011; Psotta and Brom, 2016), other studies did not confirm its three-factor structure in preschool children (Schulz et al., 2011; Hua et al., 2013; Kokštejn et al., 2018). In line with our findings, Schulz et al. (2011) found a one-factor structure supporting the notion of a general motor ability in preschool children (Hands et al., 2018). It remains to be seen whether the factor structure proposed by Henderson et al. (2007) is an appropriate representation of the motor construct in 3- to 5-year-old children. Therefore, we suggest that researchers and professionals in the clinical field should carefully interpret the separate components of young children’s MABC-2 performance. In addition, it is recommended to investigate the factor structure of motor skills and EF in a larger sample and examine its invariance across age and gender.

The current study found support for a two-factor structure of EFs consisting of performance-based EFs and rating-based EFs. These findings are difficult to compare to previous studies as previous studies did not include rating-based EF measures in their studies about the structure of EFs (Wiebe et al., 2008; Shing et al., 2010; Willoughby et al., 2010, 2012; Fuhs and Day, 2011; Masten et al., 2012; Miller et al., 2012; Usai et al., 2014; Monette et al., 2015). The non-significant to significantly moderate relationships between performance-based and rating-based EFs that have been found in earlier studies may imply that these types of measures provide different information regarding preschool children’s EFs (Miranda et al., 2015; O’Meagher et al., 2019; Tamm and Peugh, 2019). Therefore, depending on what aspect of EFs is intended to be examined, it is important to make well-considered decisions regarding the choice of an EF measure. In order to provide a comprehensive picture of EFs in preschool children, it is useful to use both types of EF measures such as the BRIEF-P (Gioia et al., 2005) and an EF task battery. Recently, standardized task batteries have been developed to assess EFs. For future research it is recommended to use such a measure, such as the Executive Function Touch (Willoughby and Blair, 2016).

## Conclusion

This study offers a comprehensive examination of task-specific and latent relationships between a range of motor skills and EFs in preschool children. Weak relationships between specific motor and EF items and weak latent relationships suggest that motor skills and EFs may be distinct developmental domains at preschool age. It remains to be seen in longitudinal studies whether the relationship between motor skills and EFs changes as a function of time.

## Data Availability Statement

The datasets generated for this study will not be made publicly available this study is part of a larger research project which is still ongoing.

## Ethics Statement

The study protocol was approved by the Ethics Review Committee of the Department of Pedagogical and Educational Sciences, Faculty of Behavioural and Social Sciences, University of Groningen. All parents gave written informed consent in accordance with the Declaration of Helsinki.

## Author Contributions

GV was responsible for the data collection, conducted the analysis, and wrote and edited the manuscript. EK was responsible for the data collection and contributed to the analysis and reviewing the manuscript. MC, AM, and SH supervised the study, and contributed to the writing and reviewing of the manuscript. All authors contributed to the article and approved the submitted version.

## Conflict of Interest

The authors declare that the research was conducted in the absence of any commercial or financial relationships that could be construed as a potential conflict of interest.
